# The amidation of poly(styrene-*alt*-maleic anhydride) *via N*,*N*-dimethylformamide decomposition

**DOI:** 10.1039/d5py01173f

**Published:** 2026-02-20

**Authors:** Michael-Phillip Smith, Lauren E. Ball, Ilanie Wessels, Bert Klumperman

**Affiliations:** a Department of Chemistry and Polymer Science, Stellenbosch University, Matieland 7602, South Africa; b Leibniz-Institut für Polymerforschung Dresden e. V., Saxony, Germany

## Abstract

Dimethylformamide (DMF), a commonly employed solvent during poly(styrene-*alt*-maleic anhydride) (SMAnh) functionalization, was noted to decompose and chemically modify the copolymer under laboratory relevant conditions. The *in situ* generation of dimethylamine resulted in modification of anhydride repeat units, highlighting an undocumented reactivity of DMF with SMAnh which could influence copolymer identity and reactivity.

Poly(styrene-*alt*-maleic anhydride) (SMAnh) is a widely utilized copolymer in coatings,^1^ compatibilizers,^2^ and covalent adaptable networks.^3,4^ Dimethylformamide (DMF) is a high boiling point (153 °C), polar-aprotic solvent often utilized to solvate SMAnh during anhydride modification.^5,6^ Although DMF is often considered an inert solvent, it has been shown to generate reactive species such as formyl and dimethylamine (HNMe_2_) groups, under laboratory relevant conditions, which ultimately function as nucleophiles towards anhydride functional groups.^5,7,8^ In a recent study by Klumperman and co-workers, it was conjectured that the SMAnh anhydride repeat units undergo modification when heated between 130–160 °C in DMF, due to the *in situ* production of dimethylamine.^9^ In this study we further explore the exposure of SMAnh to DMF under various conditions, in order to elucidate the extent and mechanism with which this purported modification occurs.

SMAnh was synthesized *via* reversible addition–fragmentation chain transfer (RAFT)-mediated copolymerization to afford a copolymer (SMAnh-DTC) with low dispersity molecular weight distribution and chemical composition (SI, Table S1).^10^ The thiocarbonylthio ω-chain end was thereafter removed *via* radical induced reduction (yielding SMAnh-H) to eliminate the contribution of end groups towards the thermal and spectroscopic profile of the copolymer (Table S1 and Fig. S1).^9^ To emulate the reaction conditions utilized by Klumperman and co-workers, SMAnh-H samples (20% (w/v)) were heated at 130 °C for 24 hours in either DMF or 1,4-dioxane (as a nonreactive control) in either a sealed vial (static headspace) or a flask fitted with a Vigreux column (mobile headspace). Modification of MAnh repeat units was tracked *via* Attenuated Total Reflectance Fourier Transform Infrared (ATR-FTIR) spectroscopy throughout the reaction, using the change in transmittance percentage (Δ*T*%) of the C=O asymmetric stretching frequency at 1857 cm^−1^ (Fig. S2 and Table 1). The type of hydrolysis or nucleophilic addition of HNMe_2_) was further characterized through observation of carbonyl stretching frequencies between 1765–1610 cm^−1^. The signal at 1642 cm^−1^ was attributed to the formation of secondary amide carbonyl groups within maleamic acid repeat units (Fig. S2 and S3). The absence of this signal, but the appearance of a C=O stretching frequency at 1726 cm^−1^ (with a corresponding OH stretching band between 3675–3122 cm^−1^) was attributed to hydrolysis of MAnh repeat units to maleic acid (MAc) repeat units (Fig. S2 and S3).

As expected, reactions conducted in 1,4-dioxane exhibited no evidence of HNMe_2_-mediated modification, but exhibited some evidence of MAnh repeat unit hydrolysis (Table 1 and Fig. S4). Conversely, reactions conducted in DMF resulted in significant modification of Manh repeat units, irrespective of the reaction vessel utilized (Fig. 1 and Fig. S2, S3). The rate of modification was significantly lower when a larger dynamic headspace was utilized, owing to the volatile nature of dimethylamine (bp. 6.8 °C). While this likely results in lower [HNMe_2_] in solution throughout the reaction, both reaction vessel configurations yielded a similar extent of modification at 24 h. SMAnh-H (a white powder) heated in DMF exhibited significant discoloration (Fig. 1). To assess whether the thermal properties of the copolymer (SMAnh-H) had been altered after heating in DMF, owing to the extensive alteration of MAnh repeat units (*i.e*. SMAnh-NMe_2_), the copolymer was analysed *via* thermogravimetric analysis (TGA). Indeed, SMAnh-NMe_2_ exhibited a higher onset degradation temperature (341 °C) than SMAnh-H (280 °C, Fig. 2A). The MAnh repeat units of SMAnh-H undergo decarboxylation from ~280 °C, a process which is notably absent in the thermogravimetric profile due to the minimal anhydride content in the SMAnh-NMe_2_ copolymer.^11^

1D and 2D NMR spectroscopy was utilized to characterize and confirm the modification of the anhydride functionality (Fig. S5–S9). Distinct differences were observed between the ^1^H NMR spectra of SMAnh-NMe_2_ compared to SMAnh-H. Signals in the aromatic region (H_d_, 5.5–7.9 ppm, Fig. S6) would suggest a significant alteration in the chemical environment surrounding the styrene repeat units had occurred. Most notably, the signal corresponding to carboxylic acid protons increased (12.3 ppm, marked with an asterisk in Fig. S6), MAnh repeat unit methine protons (H_a_) decreased in intensity and a broad signal appeared in the region of 2.8 ppm which was attributed tentatively to the methyl protons (H_f_) of maleamic acid repeat units. HSQC NMR spectroscopy was conducted for SMAnh-H (Fig. S6) and SMAnh-NMe_2_ (Fig. S7) in order to assign signals to maleamic acid units with higher confidence. The HSQC NMR spectrum for SMAnh-NME_2_ (Fig. S7) clearly indicates the absence of signals corresponding to MAnh repeat units (C/H_e+f_ in Fig. S6) and the presence of signals for maleamic acid repeat units (C/H_k_, Fig. 2C & S7). Furthermore, ^13^C NMR spectroscopic analysis of SMAnh-NME_2_ (130 °C DMF, Fig. 2B & S8) shows a clear downfield shift of carbonyl and aromatic quaternary carbons (C_g_ and C_c_, respectively), as well as the appearance of a shoulder upfield of the phenyl carbons (C_d_) upon amidation of MAnh repeat units. The changes in chemical shifts of the phenyl carbons could be indicative of styrene pendant groups adjacent to the secondary amide pendant group of maleamic acid repeat units. Notably, the ^13^C NMR spectra of SMAnh-H before and after heating in 1,4-dioxane could not be distinguished (Fig. S8), reiterating the role played by DMF in the observed alteration of the SMAnh copolymer. SMAnh-NME_2_ was also analysed *via* DOSY NMR spectroscopy, which indicated that methyl protons assigned to maleamic acid units (H_k_, Fig. S7) had similar diffusion coefficients (2.0–2.4 × 10^−11^ m^2^ s^−1^) compared to styrenic protons (H_d_, 2.0 × 10^−11^ m^2^ s^−1^, Fig. S9). Overall, the ATR-FTIR and NMR spectroscopic analyses of SMAnh-H before and after heating in DMF suggest that significant modification of anhydride groups occurs *via* the nucleophilic addition of dimethylamine to MAnh repeat units.

DMF decomposes slightly at its boiling point to afford small quantities of dimethylamine and carbon monoxide, whereby this decomposition can be exacerbated at elevated temperature (or made possible at ambient temperature) using a catalytic acid/base.^8,12^ Inspection of the DMF utilized (anhydrous, >99.8% purity, Merck Life sciences) *via* ^1^H and ^13^C NMR spectroscopy indicated the absence of water, but the presence of dimethylamine (0.5%) (Fig. S10). While further decomposition of some DMF is possible during the reaction, the minimal quantity of dimethylamine available is likely insufficient for the near quantitative modification of anhydride units observed. Despite the expansive utility of DMF in organic synthesis (as a solvent, reagent, catalyst or stabilizer) the DMF-mediated functionalization of anhydrides is rarely reported.^5^ In one instance the preparation of *N*,*N*,*N*′,*N*′-tetramethyl succinamide was reported, which required the use of catalytic amounts of sulfuric acid to facilitate the *in situ* production of HNMe_2_ from DMF.^8,13^ Inspection of the SMAnh utilized *via* ^1^H NMR spectroscopy indicated that acid impurities (hydrolysed MAnh repeat units, 12.3 ppm) were present in small quantities (Fig. S5), which could in principle catalyse the decomposition of DMF.

To explore this possibility, SMAnh (at 20% w/v in DMF) was stirred at ambient temperature or 130 °C using a reflux setup in the presence/absence of excess HCl-NMe_2_ (with/without added TEA) (Fig. 3A). No appreciable modification of SMAnh was observed when treated with HCl-NMe_2_ at ambient temperature, but after deprotonation with TEA the dimethylamine produced had sufficient nucleophilicity to rapidly react with anhydride groups (Fig. S8). This resulted in near quantitative formation of SMAnh-NMe_2_ within 15 min (Fig. 3A). Notably, the intentional modification of SMAnh with TEA/HCl-NMe_2_ yielded a copolymer with a similar ^13^C NMR spectrum to SMAnh heated in DMF at 130 °C (Fig. 2B & S8). While HNMe_2_ reacts rapidly with anhydride units at ambient temperature (Fig. 3A, S8 & S16), no appreciable modification of anhydride units was observed at ambient temperature (red data, Fig. 3A) in the presence of the naturally abundant HNMe_2_ (0.5%) or acid impurities (Fig. S10 & S5). However, when the reaction was conducted at 130 °C, extensive HNMe_2_-mediated modification (95%) was observed (Fig. 3A, S8 & S12), suggesting that efficient carboxylic acid-catalysed decomposition of DMF requires the use of elevated temperatures. The effect of temperature on the rate of SMAnh modification in DMF was also demonstrated in Fig. S17, whereby the reaction temperature was increased from 100 to 160 °C, resulting in a corresponding increase in reaction rate.

The concentration of SMAnh was varied between 10–40% (w/v), where all copolymers isolated at 24 h had been modified to a similar extent (88–95%, Fig. 3B & Fig. S11–S14). Despite the concentration of the initial acid impurities increasing with the utilization of higher [SMAnh-H], a corresponding increase in rate of modification was not observed, which could suggest only a catalytic amount of acid is required. The reaction with the lowest SMAnh concentration underwent modification the fastest, likely due to the higher concentration of DMF utilized which causes a larger initial amount of HNMe_2_, subsequently catalysing the production of more HNMe_2_. Thus, the mechanism by which acid functional groups catalyse the decomposition of DMF throughout the reaction is presented in Scheme 1, whereby the acid functionality could derive from maleic/maleamic acid repeat units.

To explore whether limitation of acid contaminants in solution would consequently limit the extent of amidation, succinic anhydride (SAnh, at 10% w/v in DMF to mimic the concentration of MAnh repeat units at 20% w/v) was heated at 130 °C for 20 h. The absence of succinic acid in the freshly sublimed SAnh was confirmed *via* ^1^H and ^13^C NMR spectroscopy (Fig. S18). After heating for 20 h, signals corresponding to 4-(dimethylamino)-4-oxobutanoic acid were observed (C/H_a–h_ and 1644 cm^−1^, Fig. S18A–D) *via* ^1^H and ^13^C NMR and ATR-FTIR spectroscopy. While acid contaminants were absent in the SAnh prior to dissolution in DMF (Fig. S18C), inspection of the 0 h kinetic sample in Fig. S18C suggests the presence of carboxylic acids at 12.3 ppm. This is likely due to rapid nucleophilic addition of the naturally abundant HNMe_2_ impurity (0.5%) in DMF to SAnh, thus producing a catalytic quantity of COOH *in situ*. Despite the absence of acid impurities in the anhydride functional species, it appears that inhibition of the process depicted in Scheme 1 is unlikely.

## Conclusions

Heating SMAnh in DMF resulted in the modification of anhydride units *via* nucleophilic addition of dimethylamine, which was generated *in situ* from the decomposition of DMF. ATR-FTIR and 1D/2D NMR spectroscopy demonstrated the conversion of anhydride units to maleamic acid/secondary amide-containing units when exposed to DMF at 130 °C (10–40% w/v). In comparison, heating in an inert solvent such as 1,4-dioxane shows minor hydrolysis under identical conditions. The kinetics of this amidation reaction could be influenced by varying the temperature or extent of dimethylamine volatilization (static *vs*. dynamic headspace). Thermal analysis indicated dissimilar degradation profiles due to the loss of anhydride units within the copolymer backbone. A comparative experiment using succinic anhydride showed that even initially acid-free anhydrides can acquire catalytic carboxylic acid units *via* the rapid addition of trace amount of HNMe_2_ present in DMF and undergo analogous amidation at 130 °C. This study reveals an unexpected reactivity of DMF with anhydride containing copolymers, whereby application of the reaction conditions employed in this study can lead to undesired alterations in copolymer chemical identity, thermal behaviour and reactivity. For researchers who employ anhydride functional materials in synthetic procedures (where retention of the anhydride is desired), precautions should be taken to avoid the use of DMF at elevated temperatures (*i.e*. using an alternative solvent, ensuring effective removal of volatile amines, *etc*.).

## Figures and Tables

**Fig. 1:**
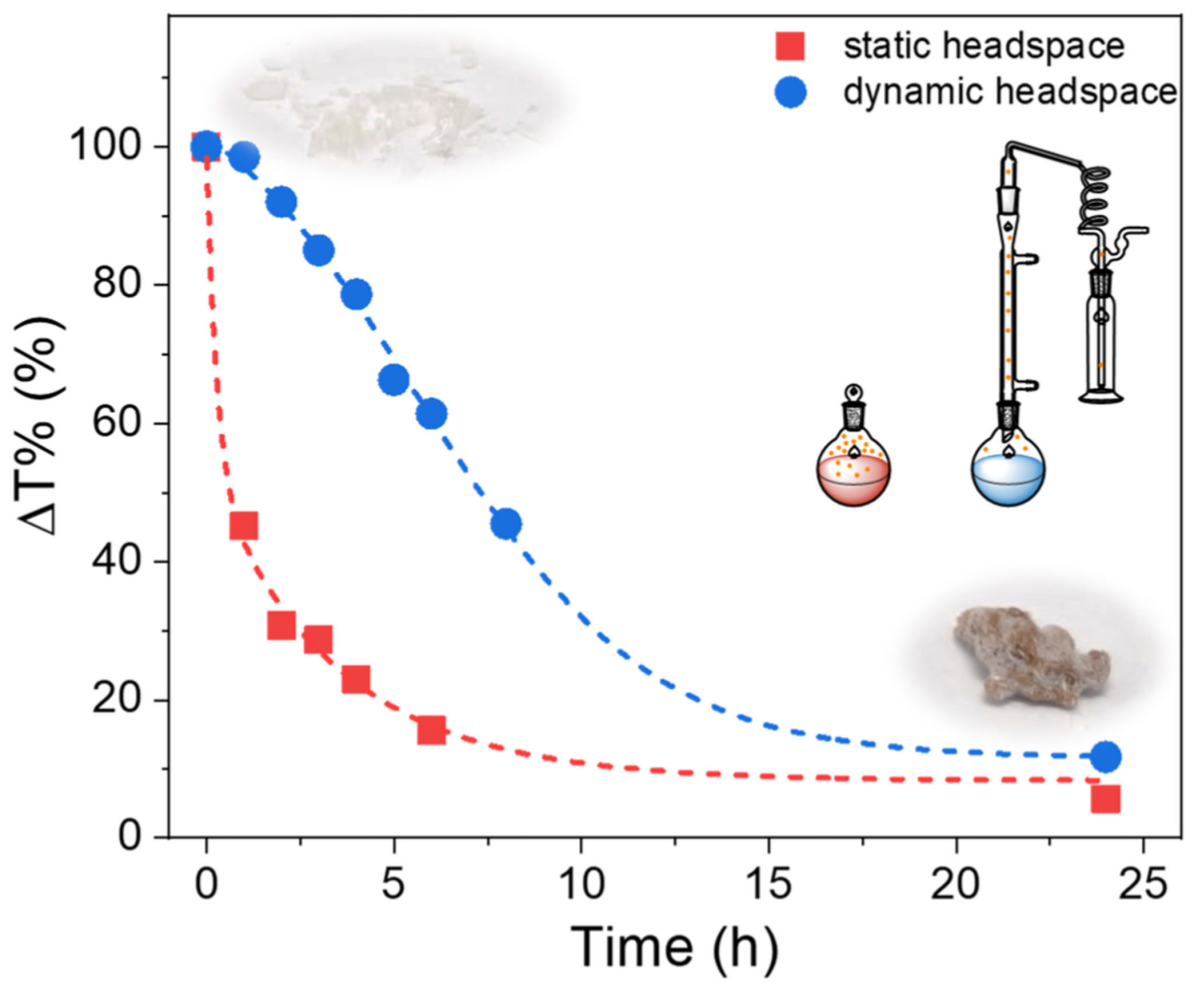
Kinetic analysis of anhydride functional group modification *via* ATR-FTIR spectroscopy, using SMAnh-H at 20% (w/v) in DMF at 130 °C in a closed and *‘*open*’* reaction vessel configuration.

**Fig. 2:**
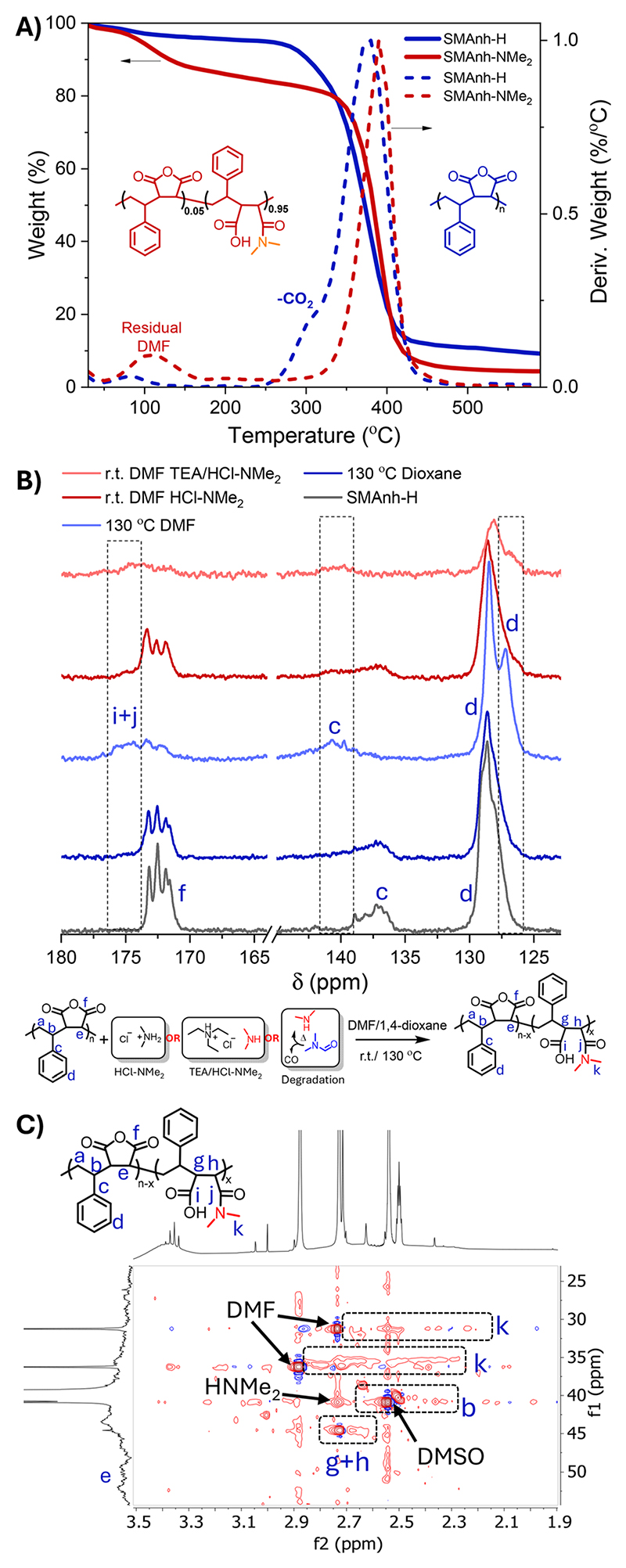
(A) TGA for SMAnh-H and SMAnh-NMe_2_, (B) ^13^C NMR spectroscopic analysis of SMAnh-H heated in dioxane/DMF at 130 °C or treated with a form of dimethylamine at ambient temperature and (C) HSQC NMR analysis of SMAnh-NMe_2_ (after heating at 130 °C in DMF).

**Fig. 3:**
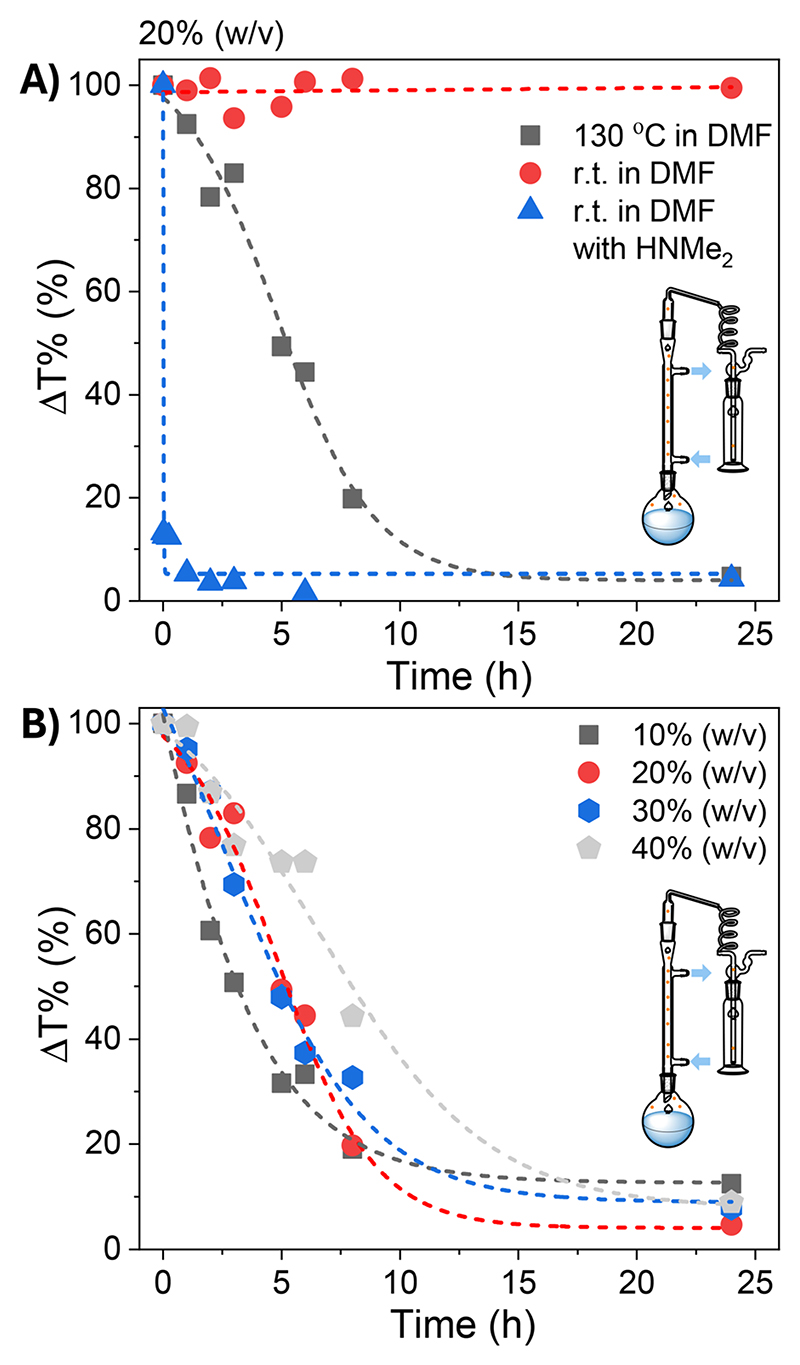
Kinetics of the modification of SMAnh at (A) (20% (w/v)) in DMF (25/130 °C), with/without added dimethylamine or (B) using varying concentrations of SMAnh (10*–*40% (w/v)) in DMF at 130 °C.

**Scheme 1:**
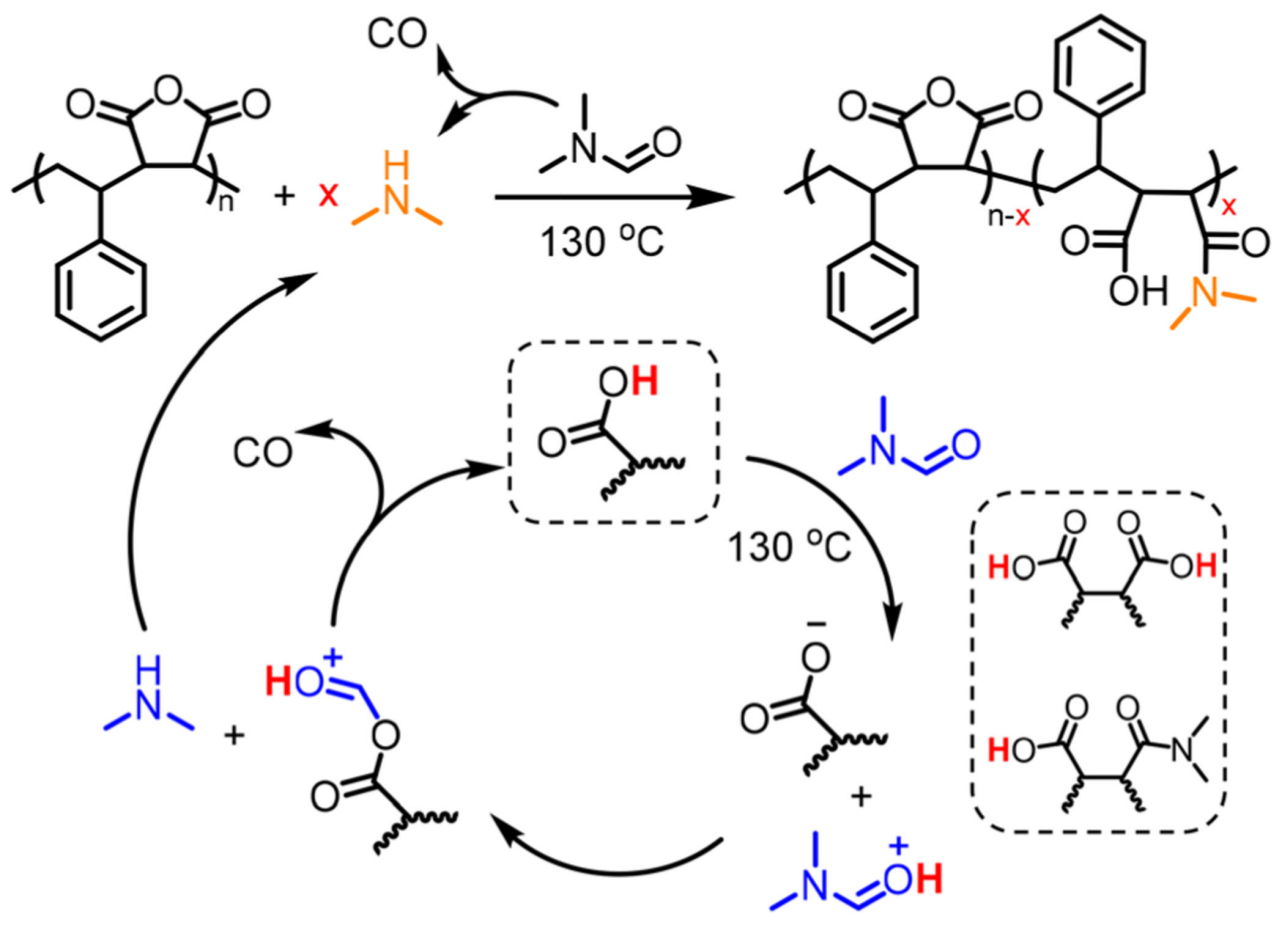
Proposed mechanism for the generation of dimethylamine, *via* the acid-catalysed decomposition of DMF at elevated temperatures.

**Table 1:** SMAnh heated in different solvents and reaction vessels, with corresponding quantitative/qualitative assessment of the modified copolymers

Entry	Solvent	Sealed (S) or reflux (R)	Δ*T*%^a^ (%)	Colour change
1	DMF	S	94	Yes (beige)
2	DMF	R	88	Yes (dark brown)
3	1,4-Dioxane	S	18	No (white)
4	1,4-Dioxane	R	14	No (white)

Determination of Δ*T*% according to eqn (S1).

## Data Availability

All the data relevant to the manuscript has been collated in the supplementary information (SI). Supplementary information is available. See DOI: https://doi.org/10.1039/d5py01173f.
