# Supplementary material for: The amidation of poly(styrene-alt-maleic anhydride) via N,N-dimethylformamide decomposition
Source: Polym Chem. 2026 Feb 20;17(10):984–8. doi: 10.1039/d5py01173f (PMC12937933; doi:10.1039/d5py01173f)
Supplement: Supplementary file 1 [file PY-017-D5PY01173F-s001.pdf]

Supplementary Information (SI) for Polymer Chemistry.
This journal is © The Royal Society of Chemistry 2026

# Supplementary information

## The amidation of poly(styrene-*alt*-maleic anhydride) *via* *N*,*N*-dimethylformamide decomposition

**Michael-Phillip Smith, Lauren Elaine Ball, Ilanie Wessels, Bert Klumperman***

## Contents

Materials ....................................................................................................... 2
Characterization ............................................................................................. 2
  Size exclusion chromatography ............................................................ 2
  Nuclear magnetic resonance (NMR) spectroscopy ................................ 2
  Attenuated Total Reflectance Fourier Transform Infrared (ATR-FTIR) spectroscopy ........ 2
  UV-Vis Spectroscopy ............................................................................. 2
  Thermogravimetric analysis (TGA) ........................................................ 2
Synthesis ....................................................................................................... 3
  Copolymer synthesis ............................................................................. 3
  Thiocarbonylthio group removal ........................................................... 3
  Varied reaction vessel configuration .................................................... 4
    Sealed vial ........................................................................................ 4
    Opened vial ...................................................................................... 4
References ..................................................................................................... 13

# Materials

Styrene (99.9%, 10-15 ppm 4-*tert*-butylcatechol inhibitor, Merck) was eluted through an aluminium oxide column thrice, 5 min before the copolymerization commenced. Maleic anhydride (99%, Merck) was purified *via* recrystallization in freshly distilled chloroform. Azobisisobutyronitrile (AIBN) was recrystallized in methanol. 1,3,5-Trioxane (>99%, Merck) was utilized as an internal standard and was purified by vacuum sublimation at 50 °C overnight in a sealed Schlenk flask. Carbon disulfide (>99.9%, Merck), (1-bromoethyl)benzene (97%, Merck), 1,1,1,3,3,3-hexamethyl-2-trimethylsilyl trisilane (97%, Merck), 3,5-dimethylpyrazole (99%, Merck), 1,4-dioxane (>99.5%, anhydrous, Merck), and *N*,*N*-dimethylformamide (DMF, 99.8%, anhydrous, Merck) were utilized as received.

# Characterization

## Size exclusion chromatography

SEC analyses utilized THF (5% (v/v) AcOH with 0.125% BHT, Merck, for HPLC, ≥99.9%) as the mobile phase with samples dissolved at 2 mg·mL$^{-1}$ and filtered using 0.45 µm RC filters (Sartorius) before analysis. All analyses were performed on an Agilent 1260 HPLC instrument fitted with a quaternary pump, a column compartment thermostated at 30 °C, a differential refractometer set at 30 °C and a diode array UV detector set at 254 and 320 nm. The columns utilized were two Agilent Technologies PLgel 5 Mixed-C columns (300 × 7.5 mm i.d.) and a PLgel 5 Guard column (50 × 7.5 mm i.d.). The flow rate during analysis was 1.0 mL·min$^{-1}$ and the injection volume per sample was 100 µL. The system was calibrated using narrow PS calibration standards with a molar mass range of 580–2.0 ×10$^{6}$ g·mol$^{-1}$.

## Nuclear magnetic resonance (NMR) spectroscopy

Samples were diluted/dissolved using DMSO-$d_6$ using a 400 MHz Ascend Bruker spectrometer. Quantitative carbon spectra were acquired *via* the utilization of a custom program using experiment C13IG with 3300 scans, a 15 second delay, and the zgig pulse program. The 2D spectra were acquired using the standard collection protocols suggested *via* the Bruker software.

## Attenuated Total Reflectance Fourier Transform Infrared (ATR-FTIR) spectroscopy

Analysis was conducted on a Thermo Scientific Nicolet iS10 Smart iTR spectrometer utilizing 128 scans (500–4000 cm$^{-1}$), with a background spectrum (64 scans) obtained prior to spectrum acquisition.

## UV-Vis Spectroscopy

Analysis was conducted on a Shimadzu UV-1800 UV spectrophotometer utilizing a sampling interval of 0.1 nm for a wavelength range of 200.00-700.00 nm. A slit width of 1.0 nm was used with a four-point operation threshold of 0.001. A background spectrum was obtained from the respective solvents and automatically subtracted prior to spectrum acquisition.

## Thermogravimetric analysis (TGA)

Analysis was performed on a TGA Q500 instrument under a flow of nitrogen gas (40.0 mL/min). Samples of 1-5 mg were placed in an aluminium pan before being subjected to a temperature ramp of 10.00 °C/min to a final temperature of 600 °C.

# Synthesis

## Copolymer synthesis

The RAFT agent utilized (1-phenylethyl 3,5-dimethyl-1*H*-pyrazole 1-carbodithioate) was synthesized according to prior literature to obtain the expected literature yield.[1] For the copolymerization, a 100 mL oven dried flask was charged with a stirrer bar, the RAFT agent (0.53 g, 1.9 mmol, 1 eq.), styrene (10 g, 96 mmol, 50 eq.), maleic anhydride (9.4 g, 96 mmol, 50 eq.), AIBN (0.06 g, 0.4 mmol, 0.2 eq.), 1,3,5-trioxane (0.02 g) and 1,4-dioxane (65 mL). The flask was thereafter sealed with a septum and sparged for 20 minutes with argon. Thereafter a 0 h kinetic sample was collected, and the flask immersed in a preheated oil bath (60 °C) for 24 h. The copolymer was isolated *via* precipitation from hexane and isolated *via* filtration to afford the copolymer with 99.9% gravimetric yield and a total monomer conversion of 98%.

## Thiocarbonylthio group removal

A 100 mL oven dried flask was charged with a stirrer bar, the dithiocarbamate functional SMAnh (SMAnh-DTC, 19 g, 1.9 mmol, 1 eq.), 1,1,1,3,3,3-hexamethyl-2-trimethylsilyl-trisilane (1.4 g, 5.6 mmol, 3 eq.), AIBN (0.31 g, 1.9 mmol, 1 eq.), and 1,4-dioxane (70 mL). The flask was sealed with a septum and sparged with argon for 20 min before immersion in a preheated oil bath (70 °C) for 24 h. The copolymer was precipitated from hexane and isolated *via* filtration to afford SMAnh-H. Thiocarbonylthio group removal was confirmed *via* UV-Vis spectroscopy.

**Table S1**. Copolymer data pertaining to monomer conversion and molecular weight prior to and after radical reduced reduction of the ω-chain end.

| Copolymer | Thiocarbonyl removal (%) | α (24 hr) | $M_n^{theo}$ (g/mol) | $M_n^{SEC}$ (g/mol) | *Đ* |
|---|---|---|---|---|---|
| SMAnh-DTC | - | 0.98 | 10 100 | 9 200 | 1.21 |
| SMAnh-H | 100 | - | 9 900 | 9 000 | 1.29 |

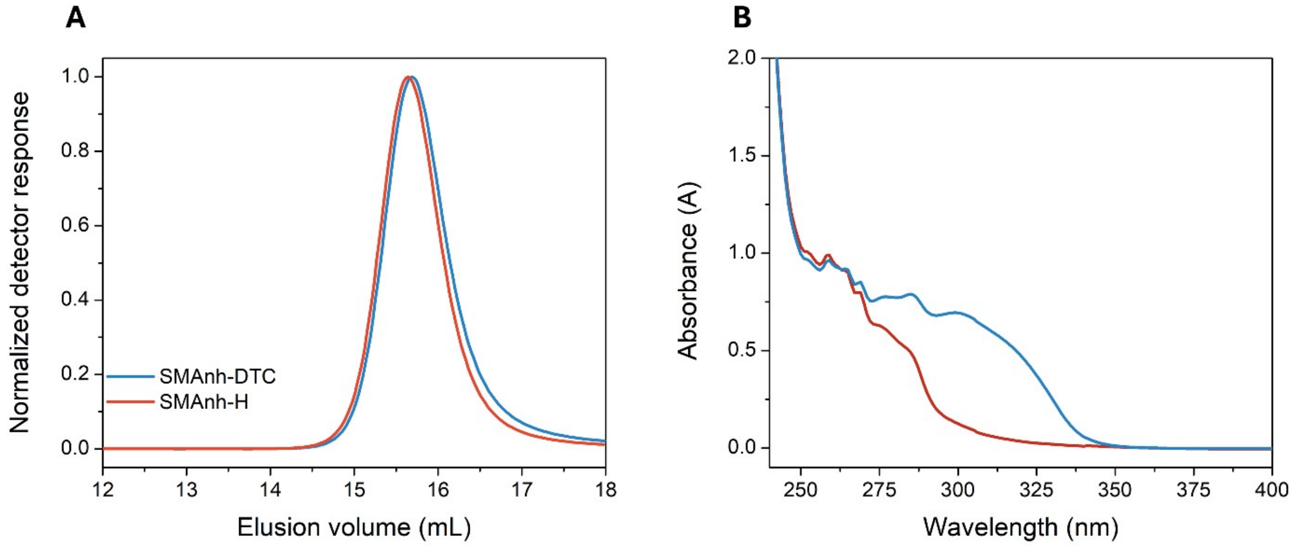


**Figure S1**. SMAnh-DTC (blue) and SMAnh-H (red) SEC analysis (A) and UV-Vis spectroscopic analysis (B). The RI eluograms confirm that the copolymer distribution was unaffected by the radical induced reduction of the thiocarbonylthio group and UV-Vis spectroscopy confirmed that SMAnh-H did not have thiocarbonylthio functional chains ends (i.e. the absence of the π-π* absorbance band at 310 nm).

## Varied reaction vessel configuration

### Sealed vial

A series of 2 mL vials were filled with argon and then charged with a stirrer bar and a 20% (w/v) solution of SMAnh-H in solvent (DMF or dioxane). The vials were sealed with a septum and secured parafilm. Thereafter the vials were immersed in a preheated oil bath (130 °C) and were removed from the oil bath at specified time intervals. The reaction was quenched by placing the vial on ice, subsequently warmed to room temperature and the copolymer then isolated *via* precipitation in cold diethyl ether. The copolymer was washed and filtered thrice before being placed in a vacuum oven at 40 °C for 24 h.

### Opened vial

A 100 mL 3-neck round bottom flask was flushed with argon and charged with a stirrer bar and a 20% (w/v) solution of SMAnh-H in solvent (DMF or 1,4-dioxane). The two side necks of the flasks were sealed with septa while the central neck was fitted with a Vigreux column connected to a bubbler. Where specified, a condenser connected to an ice bath and fitted with a bubbler, was utilized instead of a Vigreux column. The entire apparatus was thereafter flushed with argon. The flask was then immersed in a preheated oil bath (130 °C) and kinetic samples were collected and isolated at specified time intervals. The copolymer was isolated *via* precipitation in cold diethyl ether, washed and filtered thrice and subsequently dried in a vacuum oven at 40 °C for 24 h.

**Equation S1**. The change in transmittance percentage for the carbonyl stretching frequency at 1857 cm$^{-1}$ (from ATR-FTIR spectra normalized to 700 cm$^{-1}$ and baseline corrected) with respect to the initial T% of SMAnh-H prior to heating.

$$\Delta T\% = \frac{100 - (T\%)_{tx}}{100 - (T\%)_{t0}}$$

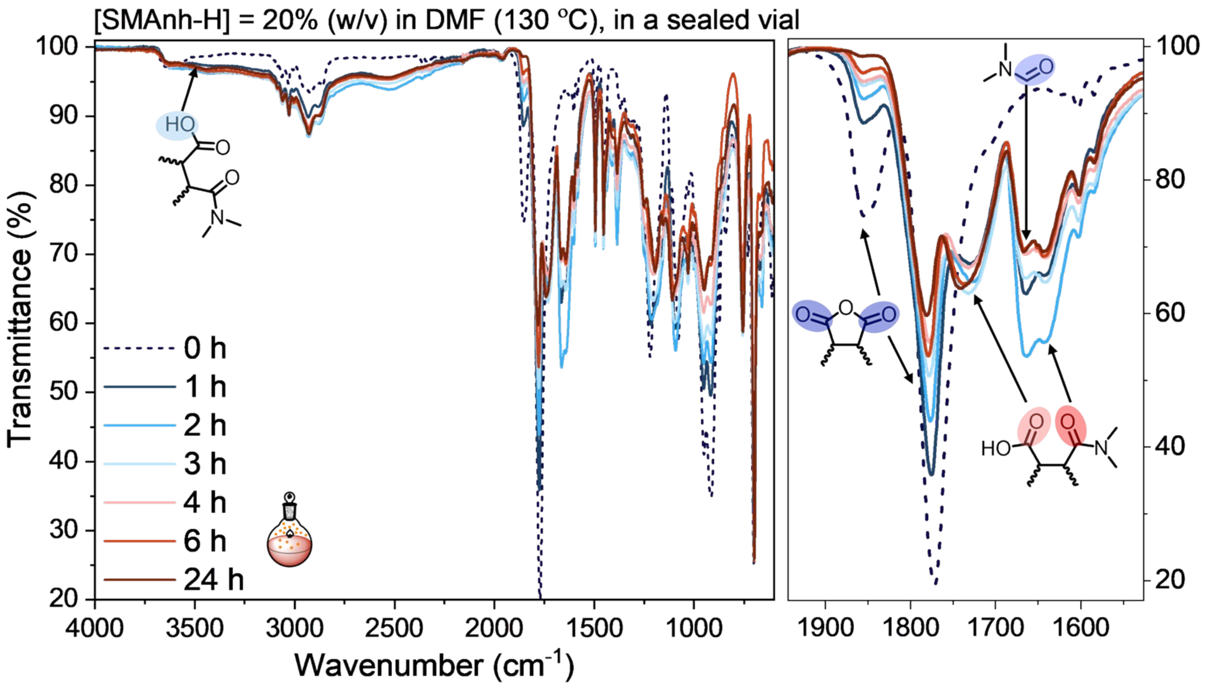


**Figure S2**. ATR-FTIR spectroscopic analysis for kinetic samples of SMAnh-H (20% (w/v)) heated at 130 °C in DMF, in a sealed reaction vessel. Signals which have been assigned to specific functional groups relevant to the reaction under investigation are highlighted.

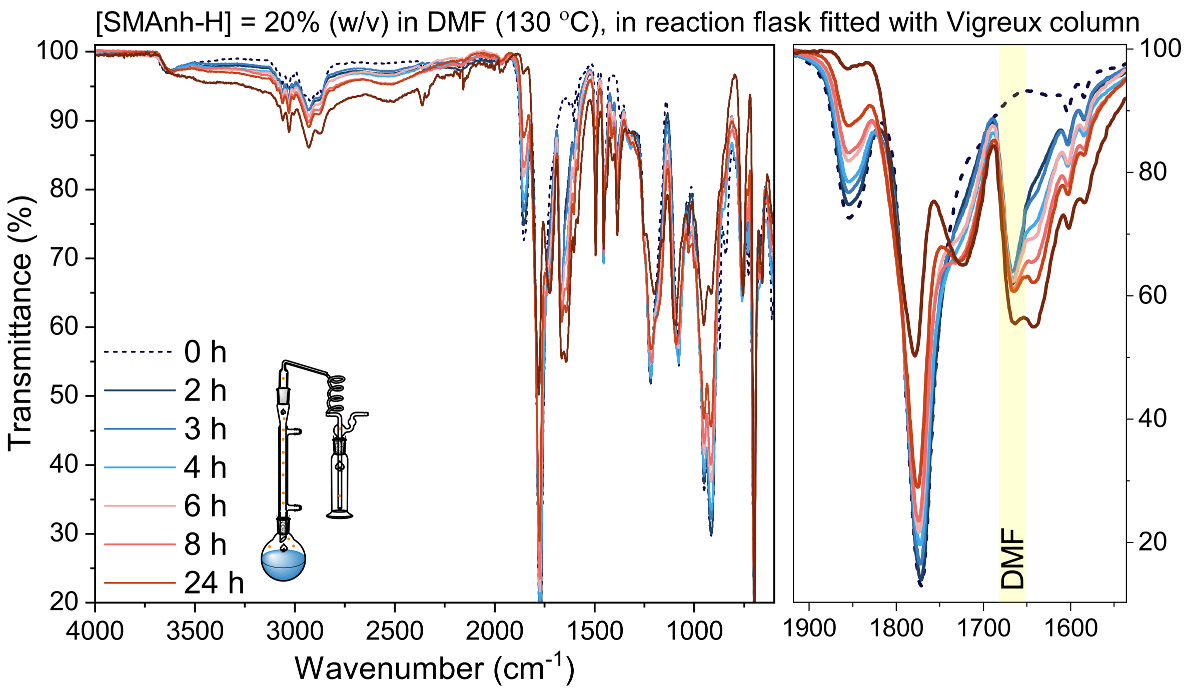


**Figure S3**. ATR-FTIR spectroscopic analysis for kinetic samples of SMAnh-H (20% (w/v)) heated at 130 °C in DMF, in a reaction vessel fitted with a Vigreux column.

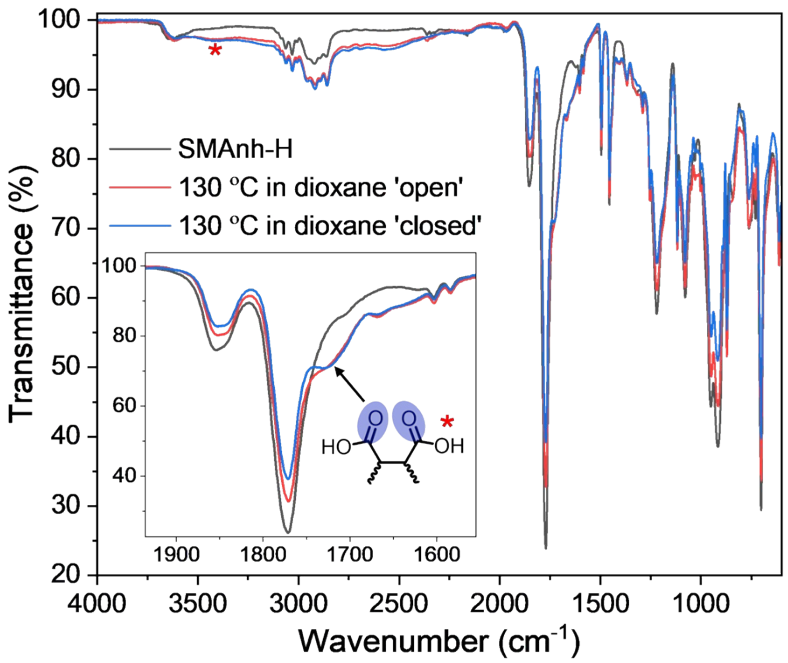


**Figure S4**. ATR-FTIR spectroscopic analysis for kinetic samples of SMAnh-H (20% (w/v)) heated at 130 °C in 1,4-dioxane, in a sealed reaction vessel or a reaction vessel fitted with a condenser.

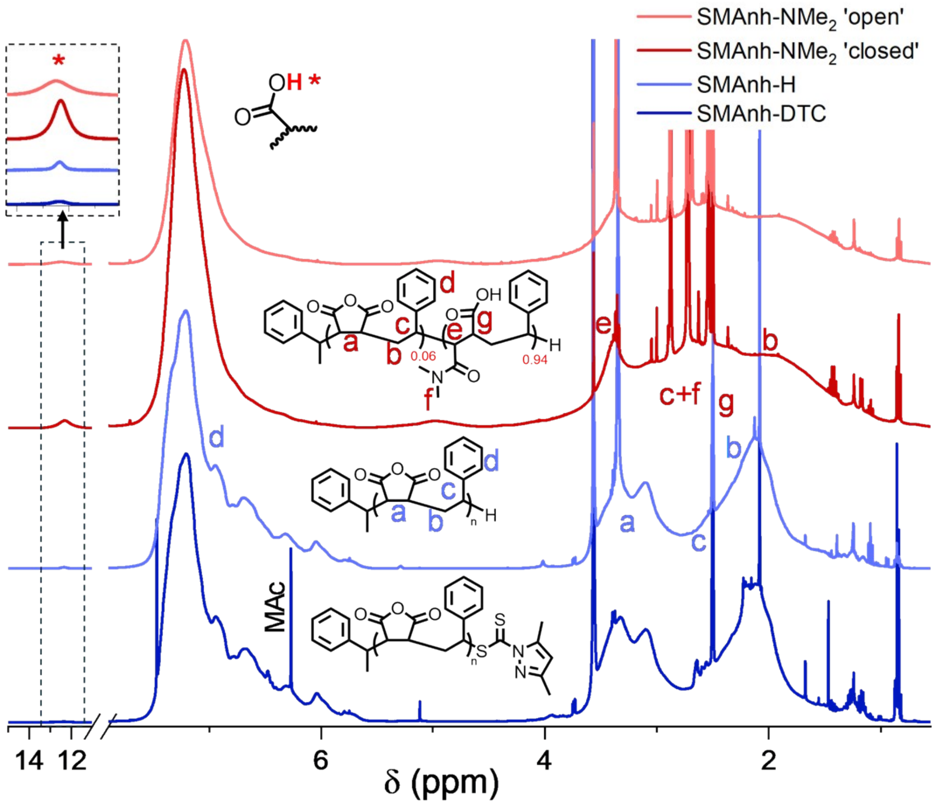


**Figure S5**. $^{1}$H NMR spectroscopic analysis (in DMSO-$d_6$) of SMAnh-DTC, SMAnh-H, and SMAnh-$NMe_2$ (derived from SMAnh heated in DMF at 130 °C for 24 h in an open/closed reaction vessel). Unlabelled protons correspond to solvent impurities such as DMF (7.95, 2.89 and 2.73 ppm), 1,4-dioxane (3.56 ppm), water (3.34 ppm), pentane (0.86 and 1.27 ppm) or a maleic acid impurity (6.27 ppm).

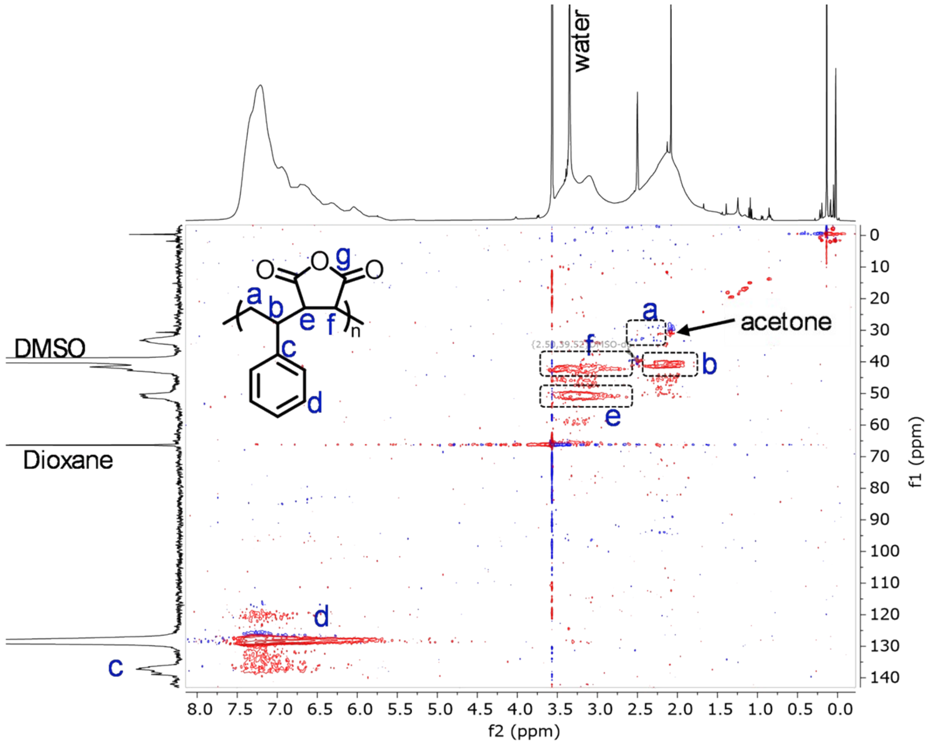


**Figure S6**. HSQC NMR spectrum for SMAnh-H in DMSO-$d_6$. The edited HSQC NMR spectrum is phased to indicate $CH_2$ protons in blue and $CH/CH_3$ protons in red. To resolve a signal for $C_a/H_a$ the intensity of the 2D plot was increased, which caused some noise in areas of the plot corresponding to the baseline. Solvent impurities are noted on the spectra.

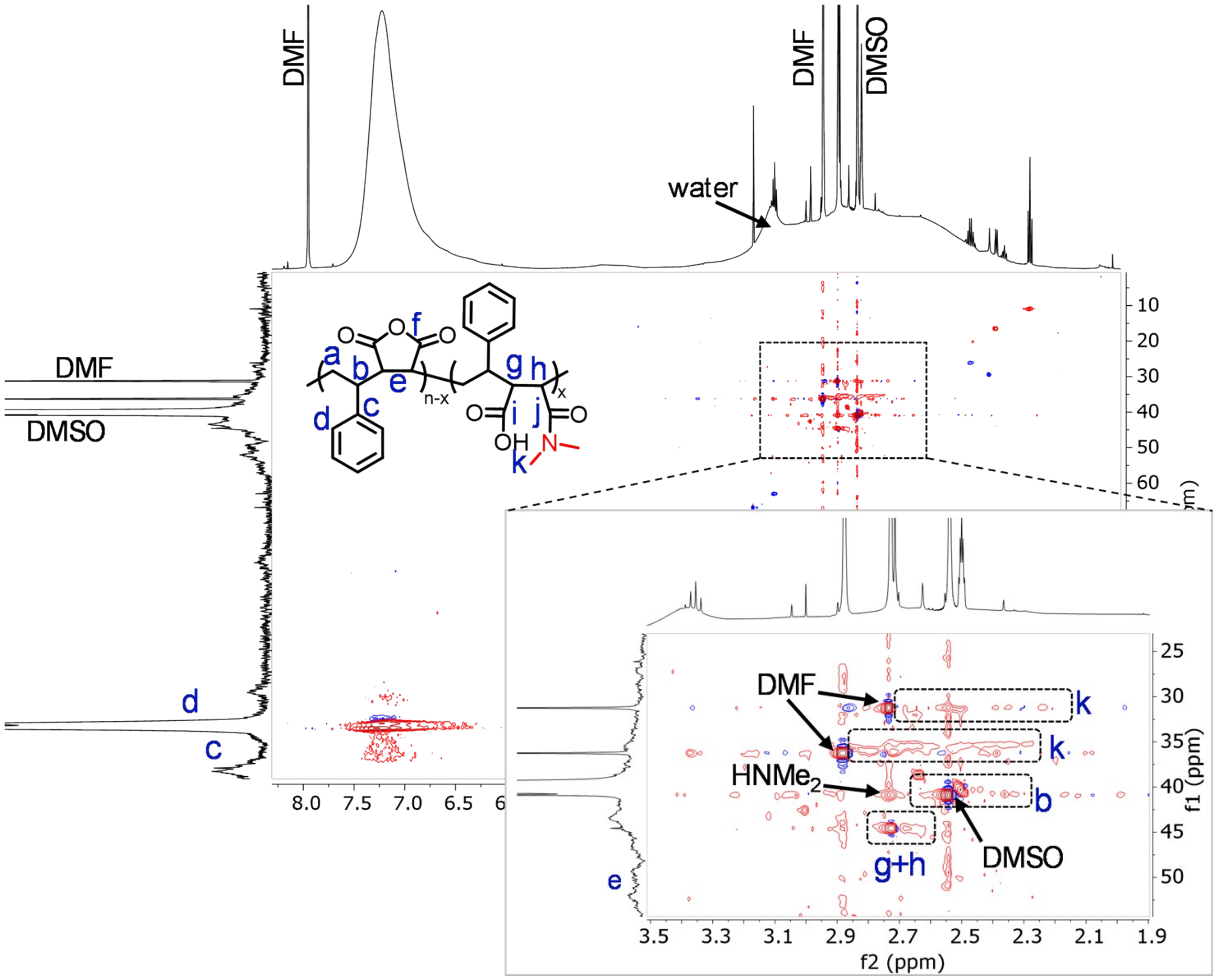


**Figure S7**. HSQC NMR spectrum for SMAnh-H (in DMSO-$d_6$) after heating in DMF at 130 °C for 24 h in a closed reaction vessel.

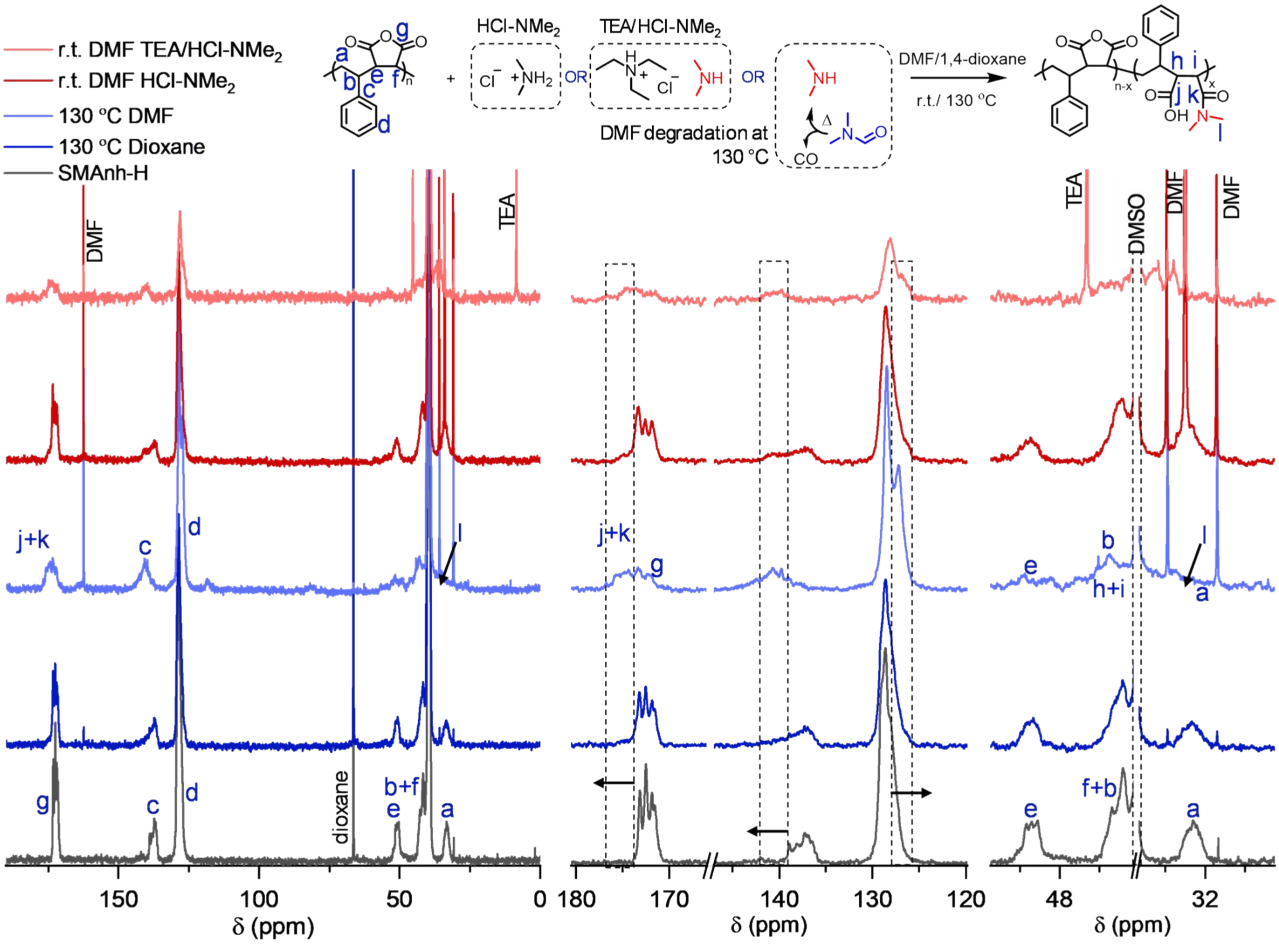


**Figure S8**. $^{13}$C NMR spectroscopic analysis (in DMSO-$d_6$) of SMAnh-H before and after heating at 130 °C in DMF/dioxane. To assign the carbons of the copolymer after heating, two comparative experiments were performed whereby SMAnh-H was treated with dimethylammonium chloride (or dimethylammonium chloride treated with triethylamine) at ambient temperature in DMF.

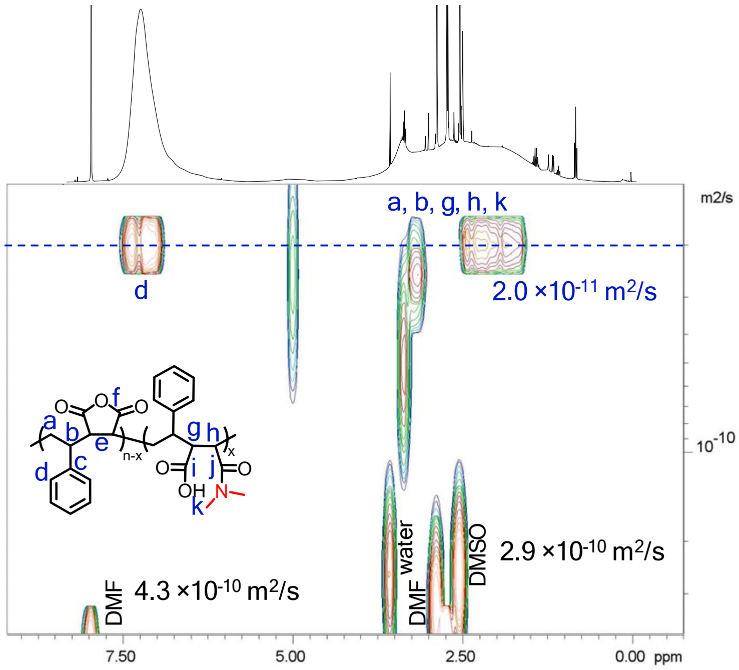


**Figure S9**. DOSY NMR analysis of SMAnh after heating in DMF at 130 °C for 24 h in a sealed reaction vessel.

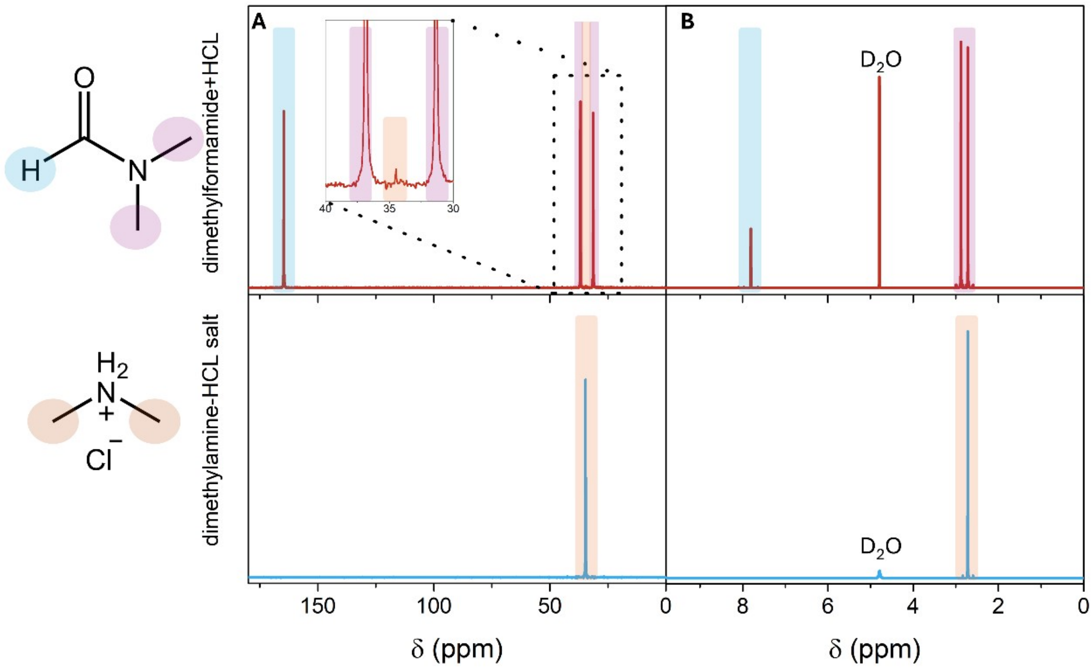


**Figure S10.** A) $^{13}$C NMR spectra and B) $^{1}$H NMR spectra of DMF treated with HCl (top) and dimethylamine-HCl salt (bottom)

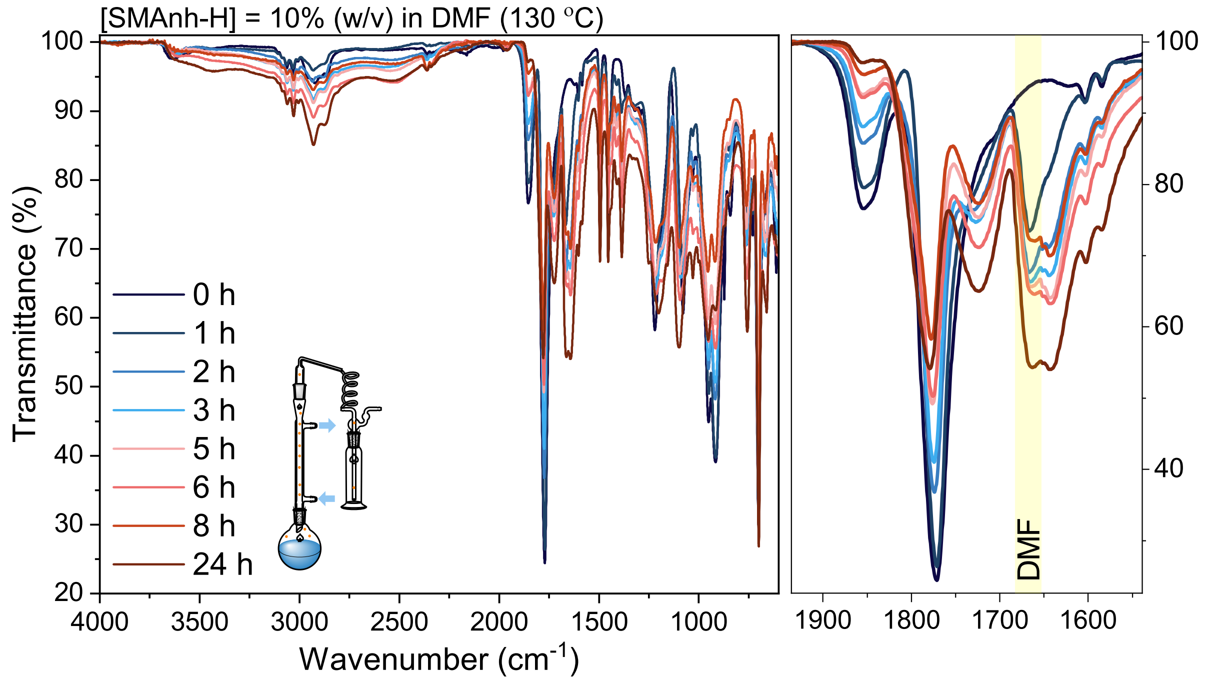


**Figure S11.** ATR-FTIR spectroscopic analysis for kinetic samples of SMAnh-H (10% (w/v)) heated at 130 °C in DMF, under reflux conditions.

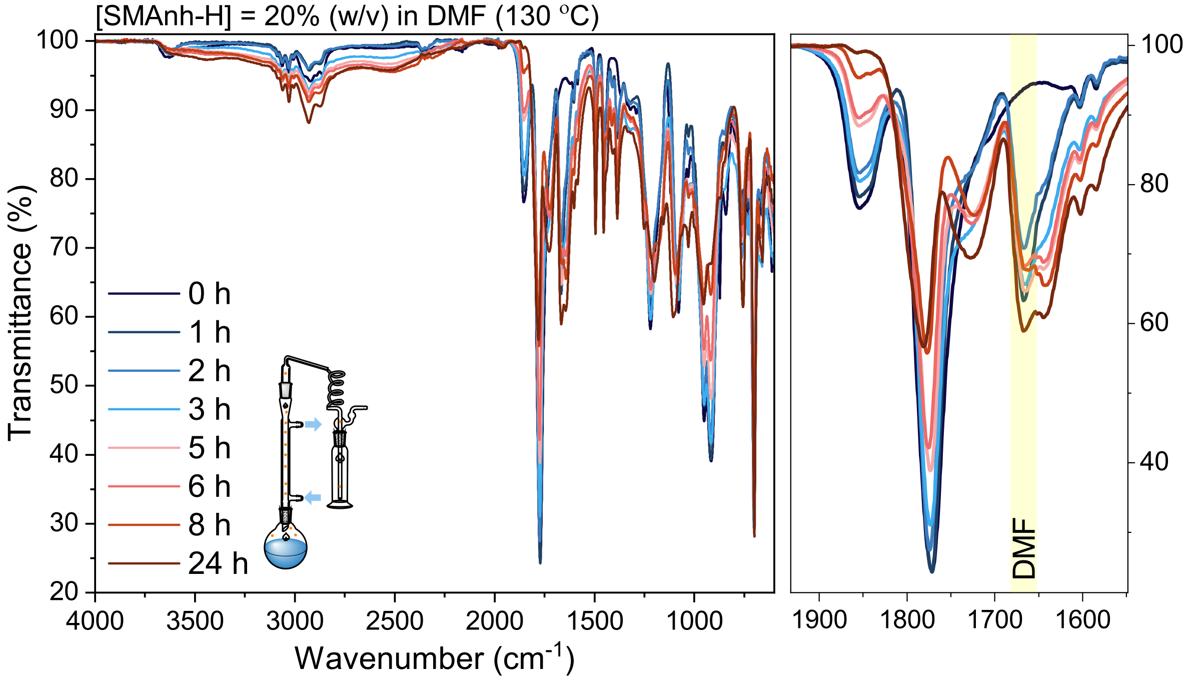


**Figure S12.** ATR-FTIR spectroscopic analysis for kinetic samples of SMAnh-H (20% (w/v)) heated at 130 °C in DMF, under reflux conditions.

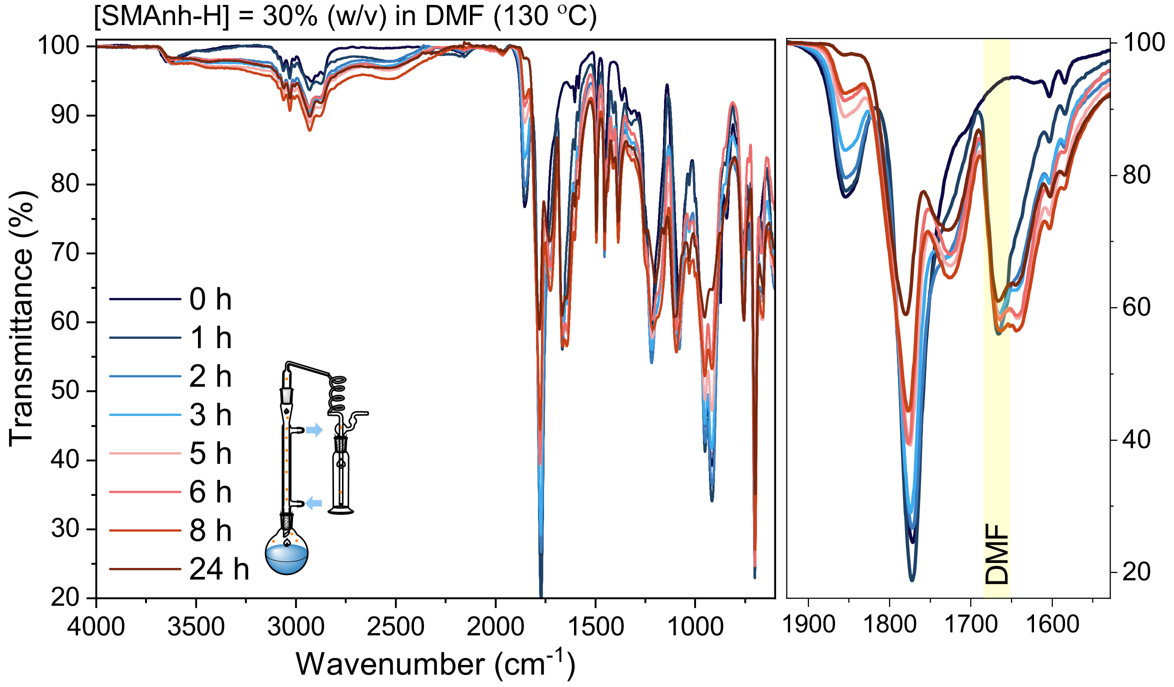


**Figure S13.** ATR-FTIR spectroscopic analysis for kinetic samples of SMAnh-H (30% (w/v)) heated at 130 °C in DMF, under reflux conditions.

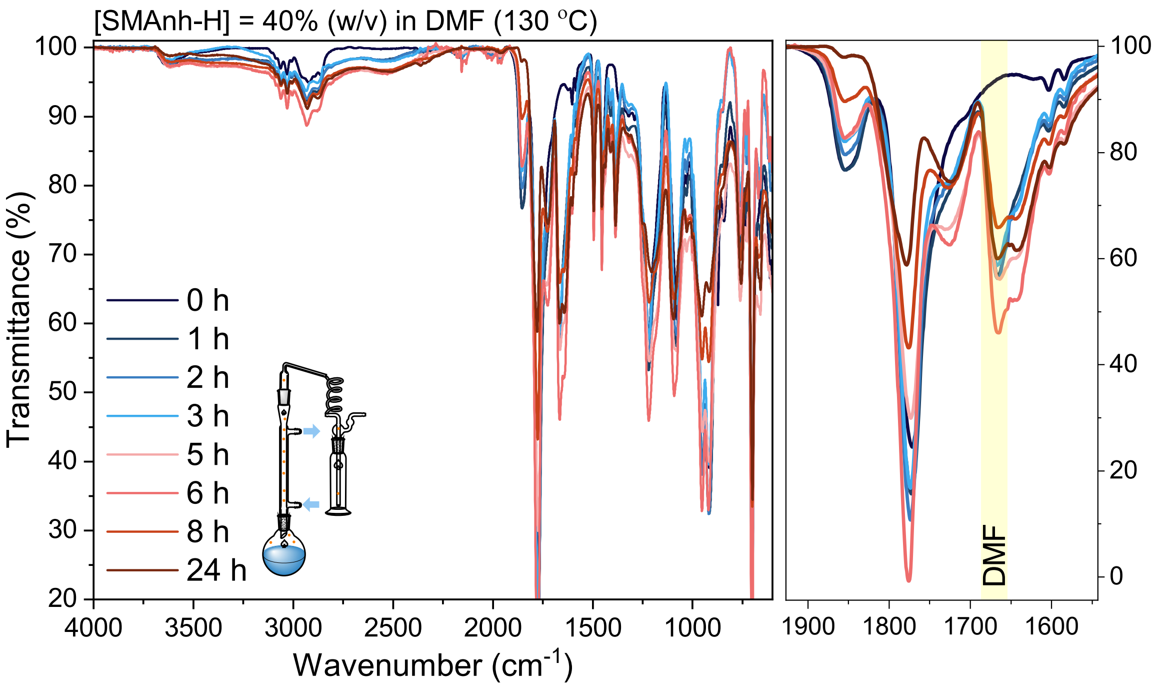


**Figure S14.** ATR-FTIR spectroscopic analysis for kinetic samples of SMAnh-H (40% (w/v)) heated at 130 °C in DMF, under reflux conditions.

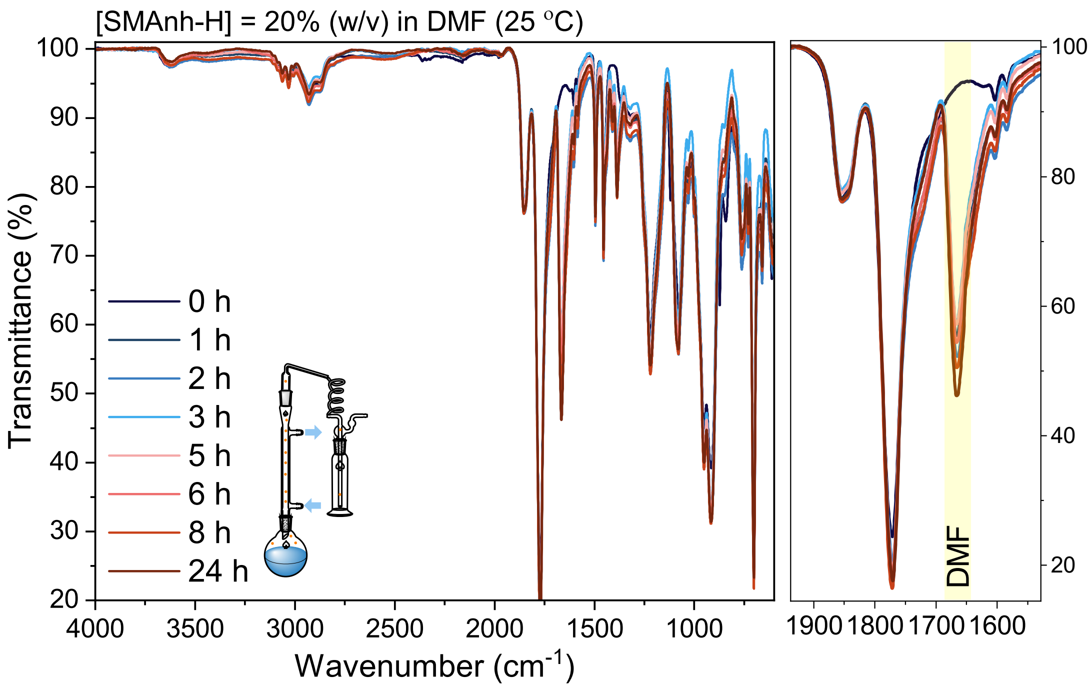


**Figure S15.** ATR-FTIR spectroscopic analysis for kinetic samples of SMAnh-H (20% (w/v)) heated at 25 °C in DMF, under reflux conditions.

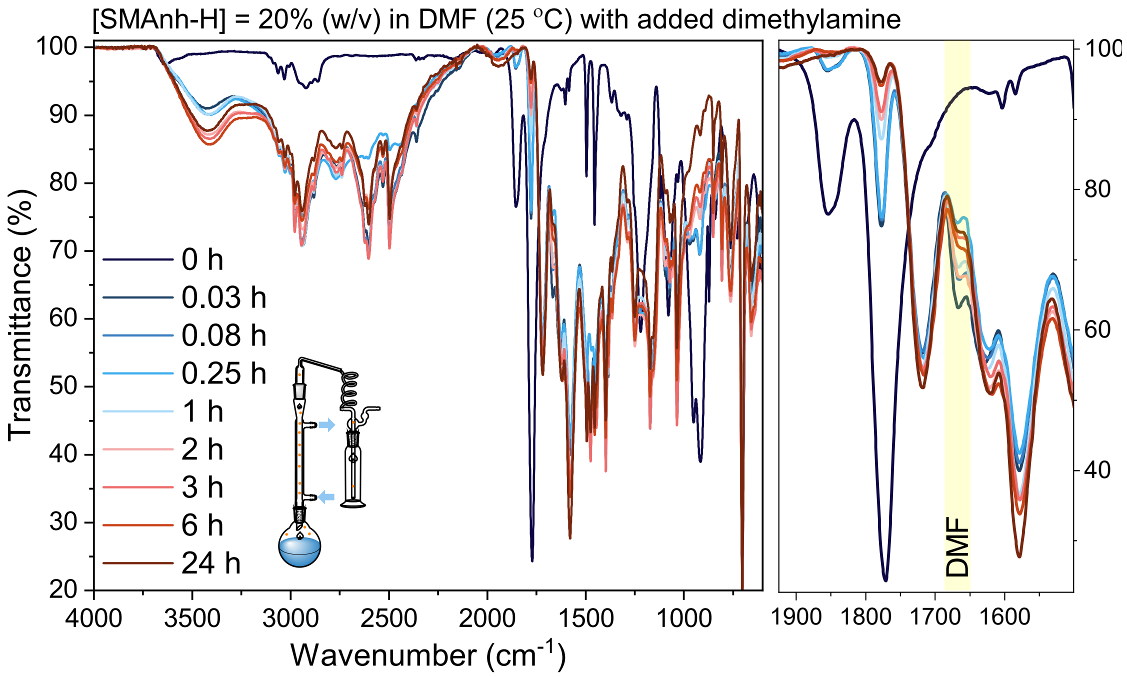


**Figure S16.** ATR-FTIR spectroscopic analysis for kinetic samples of SMAnh-H (20% (w/v)) heated at 25 °C in DMF in the presence of excess dimethylamine (deprotonated with triethylamine), under reflux conditions.

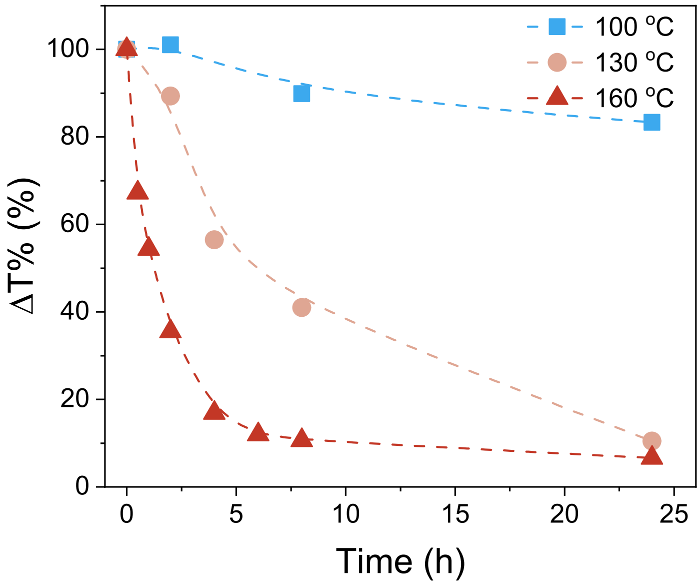


**Figure S17.** Kinetics of the modification of SMAnh at 20% (w/v) in DMF with varying reaction temperature (100–160 °C).

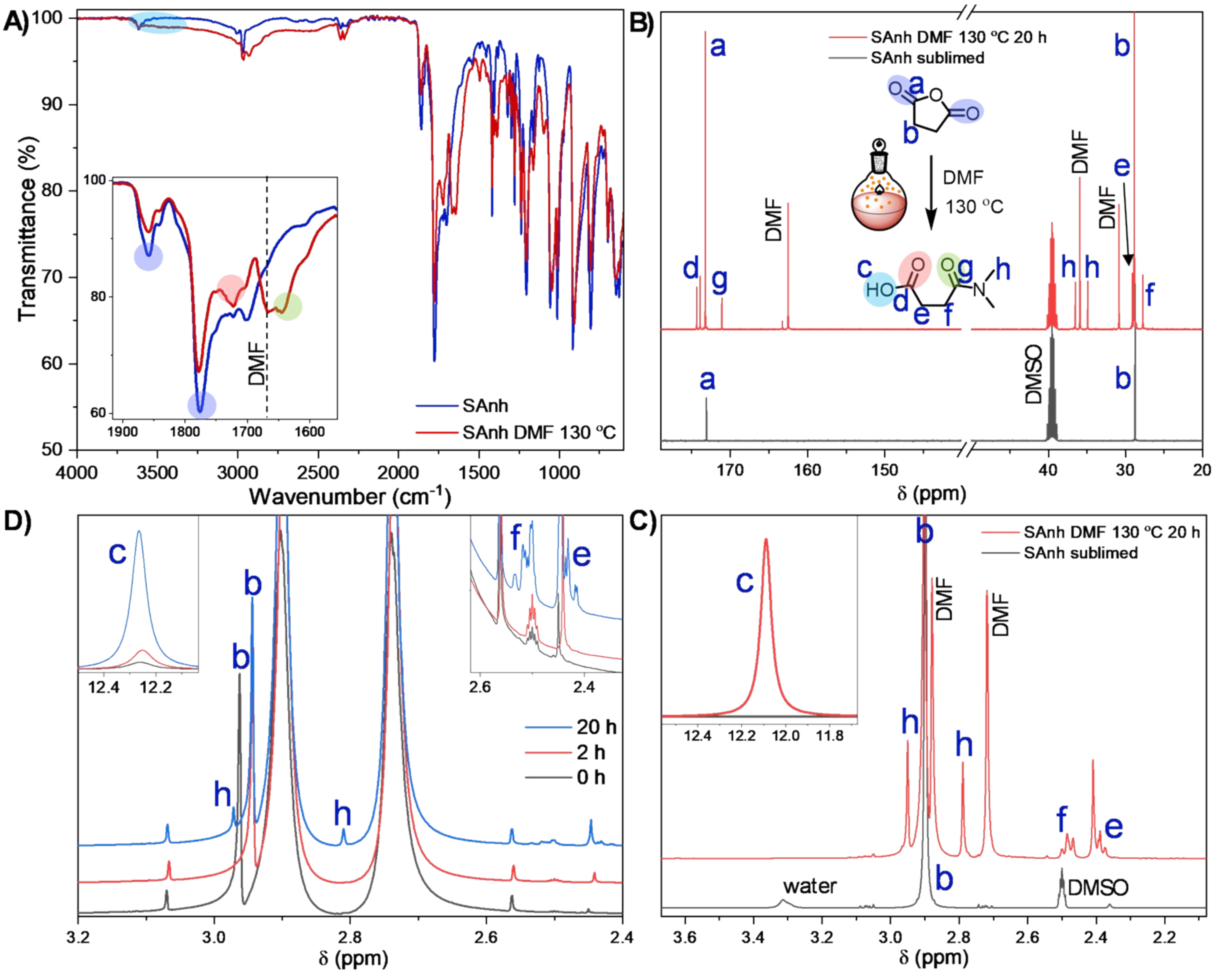


**Figure S18**. ATR-FTIR spectroscopic (A); $^{13}$C NMR (B) and $^{1}$H NMR spectroscopic analysis (C–D) of succinic anhydride (SAnh) heated in DMF at 130 °C for 20 h (analysed in DMSO-$d_6$). Kinetic samples withdrawn throughout the reaction are presented in B while the dried product is presented in C.

# References

1. L. E. Ball, M.-P. Smith and B. Klumperman, *Polym. Chem.*, 2025, **16**, 1019-1023. DOI: 10.1039/D4PY01227E.
